# Notification that new names of prokaryotes, new combinations and new taxonomic opinions have appeared in volume 75, part 1 of the IJSEM

**DOI:** 10.1099/ijsem.0.006689

**Published:** 2025-04-30

**Authors:** Aharon Oren, Markus Göker

**Affiliations:** 1 The Institute of Life Sciences, The Hebrew University of Jerusalem, The Edmond J. Safra Campus, 9190401 Jerusalem, Israel; 2 Leibniz Institute DSMZ – German Collection of Microorganisms and Cell Cultures, Inhoffenstrasse 7B, 38124 Braunschweig, Germany

This listing of names of prokaryotes published in a previous issue of the *IJSEM* is provided as a service to microbiology to assist in the recognition of new names and new combinations. This procedure was proposed by the Judicial Commission [1]. The names given herein are listed according to the rules of priority (i.e. the time and date of publication of the articles on the journal’s platform and the order of valid publication of names in the original articles) [2].

**Table:** 

**Order** of article publication	Name/author	Proposed as	Article no.	Page no.
1	*Pseudonocardia spirodelae* Butdee *et al*. 2025	sp. nov.	006608	9
1	*Pseudonocardia alni* subsp. *alni* (Evtushenko *et al*. 1989) Butdee *et al*. 2025	comb. nov. [basonym: *Amycolata alni* Evtushenko *et al*. 1989]	006608	10
1	*Pseudonocardia alni* subsp. *carboxydivorans* (Park *et al*. 2008) Butdee *et al*. 2025	comb. nov. [basonym: *Pseudonocardia carboxydivorans* Park *et al*. 2008]	006608	10
2	*Dyella aluminiiresistens* Li *et al*. 2025	sp. nov.	006611	4
3	*Tenacibaculum platacis* Lopez *et al*. 2025	sp. nov.	006605	6
3	*Tenacibaculum vairaonense* Lopez *et al*. 2025	sp. nov.	006605	8
3	*Tenacibaculum polynesiense* Lopez *et al*. 2025	sp. nov.	006605	8
4	*Luteimonas salinilitoris* Zhang *et al*. 2025	sp. nov.	006630	7
5	*Tistrella arctica* Zhang *et al*. 2025	sp. nov.	006627	7
6	*Mycobacterium wendilense* Iversen *et al*. 2025	sp. nov.	006620	10
6	*Mycobacterium burgundiense* Iversen *et al*. 2025	sp. nov.	006620	10
6	*Mycobacterium kokjensenii* Iversen *et al*. 2025	sp. nov.	006620	10
6	*Mycobacterium holstebronense* Iversen *et al*. 2025	sp. nov.	006620	10
7	*Leptospira mgodei* Hamond *et al*. 2025	sp. nov.	006595	8
7	*Leptospira iowaensis* Hamond *et al*. 2025	sp. nov.	006595	8
7	*Leptospira cinconiae* Hamond *et al*. 2025	sp. nov.	006595	8
7	*Leptospira milleri* Hamond *et al*. 2025	sp. nov.	006595	8
7	*Leptospira gorisiae* Hamond *et al*. 2025	sp. nov.	006595	8
8	*Paenibacillus zeirhizosphaerae* Márton *et al*. 2025	sp. nov.	006628	7
9	*Pseudomonas fungipugnans* Schnyder *et al*. 2025	sp. nov.	006624	10
10	*Methylobacterium litchii* Gao *et al*. 2025	sp. nov.	006639	6
10	*Methylobacterium guangdongense* Gao *et al*. 2025	sp. nov.	006639	7
10	*Methylorubrum subtropicum* Gao *et al*. 2025	sp. nov.	006639	8
11	*Pseudoalteromonas qingdaonensis* Lin *et al*. 2025	sp. nov.	006625	5
12	*Roseomonas cutis* Son *et al*. 2025	sp. nov.	006617	9
13	*Pseudomonas spirodelae* Vandeborne *et al*. 2025	sp. nov.	006637	6
14	*Owariibacterium* Hamaguchi *et al*. 2025	gen. nov.	006636	9
14	*Owariibacterium komagatae* Hamaguchi *et al*. 2025	sp. nov.	006636	9
14	*Desulfovibrio falkowii* Hamaguchi *et al*. 2025	sp. nov.	006636	11
14	*Porphyromonas miyakawae* Hamaguchi *et al*. 2025	sp. nov.	006636	12
14	*Mediterraneibacter flintii* Hamaguchi *et al*. 2025	sp. nov.	006636	13
15	*Veillonella absiana* Bai *et al*. 2025	sp. nov.	006643	7
16	*Pseudomonas retamae* Selami *et al*. 2025	sp. nov.	006646	15
17	*Clostridium sediminicola* Hirano *et al*. 2025	sp. nov.	006641	8
18	*Chryseobacterium salviniae* Sparks *et al*. 2025	sp. nov.	006644	6
19	*Paradevosia tibetensis* (Wang *et al*. 2015) Li 2025	comb. nov. [basonym: *Youhaiella tibetensis* Wang *et al*. 2015]	006634	1
20	*Jannaschia maritima* Feng *et al*. 2025	sp. nov.	006645	7
21	*Scrofimicrobium appendicitidis* Lao *et al*. 2025	sp. nov.	006633	10
22	*Streptococcus suivaginalis* Nicholson *et al*. 2025	sp. nov.	006631	9
22	*Streptococcus iners* Nicholson *et al*. 2025	sp. nov.	006631	10
22	*Streptococcus iners* subsp. *iners* Nicholson *et al*. 2025*	comb. nov. [basonym: *Streptococcus iners* Nicholson *et al*. 2025]	006631	10
22	*Streptococcus iners* subsp. *hyiners* Nicholson *et al*. 2025*	subsp. nov.	006631	10
23	*Holzapfeliella saturejae* Alcántara *et al*. 2025	sp. nov.	006654	8
24	*Flavobacterium aerium* Daussin *et al*. 2025	sp. nov.	006647	7
25	*Paludibacillus* He *et al*. 2025	gen. nov.	006655	7
25	*Paludibacillus litoralis* He *et al*. 2025	sp. nov.	006655	7
26	*Lentilactobacillus terminaliae* Phuengjayaem *et al*. 2025	sp. nov.	006649	8
27	*Desulfosporosinus paludis* Dyksma *et al*. 2025	sp. nov.	006648	7
28	*Bacillus bruguierae* Liu *et al*. 2025	sp. nov.	006656	7
28	*Bacillus kandeliae* Liu *et al*. 2025	sp. nov.	006656	9
28	*Bacillus yunxiaonensis* Liu *et al*. 2025	sp. nov.	006656	10
28	*Metabacillus rhizosphaerae* Liu *et al*. 2025	sp. nov.	006656	10
28	*Metabacillus sediminis* Liu *et al*. 2025	sp. nov.	006656	11
28	*Psychrobacillus mangrovi* Liu *et al*. 2025	sp. nov.	006656	11
29	*Flavobacterium zubiriense* Matos *et al*. 2025	sp. nov.	006660	6
29	*Flavobacterium magnesitis* Matos *et al*. 2025	sp. nov.	006660	11
30	*Ascidiimonas meishanensis* Wang *et al*. 2025	sp. nov.	006653	7
30	*Leptobacterium meishanense* Wang *et al*. 2025	sp. nov.	006653	9
31	*Comamonas halotolerans* Park *et al*. 2025	sp. nov.	006665	5
32	*Corynebacterium mayonis* Mohammed *et al*. 2025	sp. nov.	006632	6
33	*Streptacidiphilus alkalitolerans* Lee *et al*. 2025	sp. nov.	006652	8
33	*Streptacidiphilus cavernicola* Lee *et al*. 2025	sp. nov.	006652	10
33	*Streptacidiphilus jeojiensis* Lee *et al*. 2025	sp. nov.	006652	10
34	*Flavobacterium arundinis* Park *et al*. 2025	sp. nov.	006667	7
34	*Flavobacterium calami* Park *et al*. 2025	sp. nov.	006667	9
34	*Flavobacterium helocola* Park *et al*. 2025	sp. nov.	006667	10
34	*Flavobacterium flavipallidum* Park *et al*. 2025	sp. nov.	006667	10

* Automatically created in accordance with Rule 40d of the International Code of Nomenclature of Prokaryotes [2].

The following taxonomic opinions (i.e. the emendation of circumscriptions or the creation of synonyms) do not affect the status of a name under the International Code of Nomenclature of Prokaryotes. These taxonomic opinions can neither be considered approved nor disapproved by the International Committee on Systematics of Prokaryotes.

**Table:** 

Name/author	Article no.	Page no.
*Pseudonocardia alni* (Evtushenko *et al*. 1989) Warwick *et al*. 1994 emend. Butdee *et al*. 2025	006608	10
*Pseudonocardia antarctica* Prabahar *et al*. 2004 pro synon. *Pseudonocardia alni* (Evtushenko *et al*. 1989) Warwick *et al*. 1994	006608	9
*Streptacidiphilus* Kim *et al*. 2003 emend. Lee *et al*. 2025	006652	10
*Suonthocola* Hitch *et al*. 2022 pro synon. *Diplocloster* Chaplin *et al*. 2022	006616	4
*Suonthocola fibrivorans* Hitch *et al*. 2022 pro synon. *Diplocloster agilis* Chaplin *et al*. 2022.	006616	4
*Thioalkalicoccus* Bryantseva *et al*. 2000 emend. Bryantseva *et al*. 2025	006657	13
*Thioalkalicoccus limnaeus* corrig. Bryantseva *et al*. 2000 emend. Bryantseva *et al*. 2025	006657	13
*Tistrella* Shi *et al*. 2003 emend. Zhang *et al*. 2025	006627	7

**Corrections to the Notification List for volume 74, part 9 of the *IJSEM*** [3]:

*Lactobacillus amylovorus* Nakamura 1981 emend. Yanana *et al*. 2024 should be *Lactobacillus amylovorus* Nakamura 1981 emend. Yamane *et al*. 2024.

*Hyphobacterium* Sun *et al*. 2017 emend. Zhao *et al*. 2014 should be *Hyphobacterium* Sun *et al*. 2017 emend. Zhao *et al*. 2024.

*Pseudohoeflea* Hyeon *et al*. 2017 emend. Yu *et al*. 2014 should be *Pseudohoeflea* Hyeon *et al*. 2017 emend. Yu *et al*. 2024.
